# Supplementary material for: MQF and buffered MQF: quotient filters for efficient storage of k-mers with their counts and metadata
Source: BMC Bioinformatics. 2021 Feb 16;22:71. doi: 10.1186/s12859-021-03996-x (PMC7885209; doi:10.1186/s12859-021-03996-x)
Supplement: Supplementary file 1 — Additional file 1. Supplementary Figures, Supplementary Table 1 and Supplementary Table 3. [file 12859_2021_3996_MOESM1_ESM.docx]

**Supplementary materials**


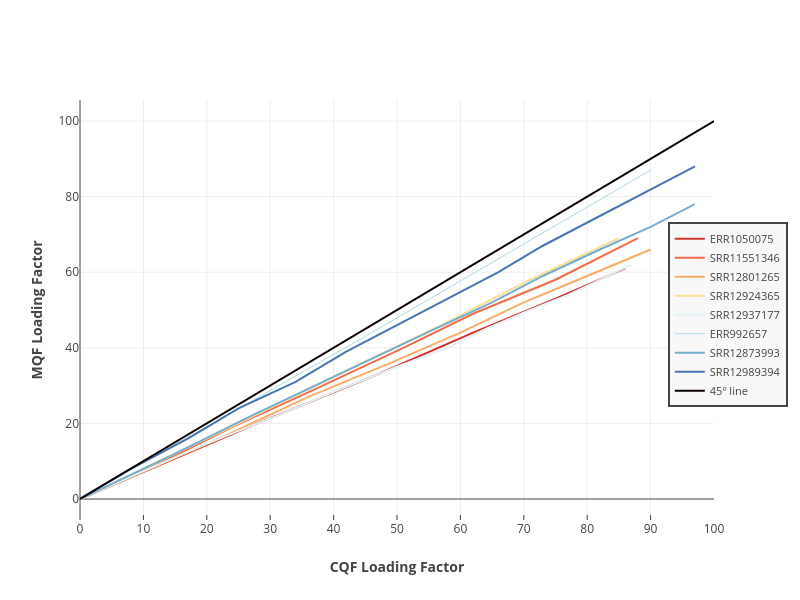


**Supplementary Figure 1: MQF has a lower load factor compared to CQF in real datasets**. Chunks of items, from 8 real datasets, were inserted iteratively into matching CQF and MQF structures. MQF had lower loading factors for all tested datasets (The further from the 45° line the better the MQF).


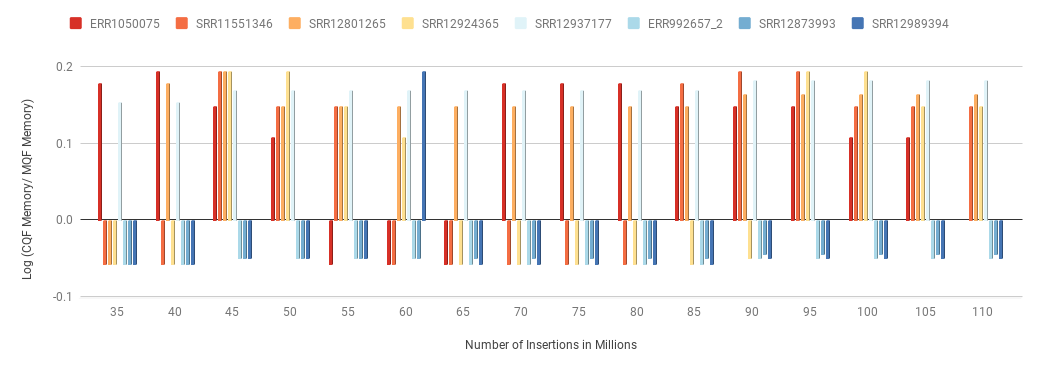


**Supplementary Figure 2: Memory consumption comparison between CQF and MQF in real datasets.** The graph compares the memory consumption of the smallest CQF and MQF that fit different datasets. The bigger the value on the y-axes, the more memory the MQF saved.


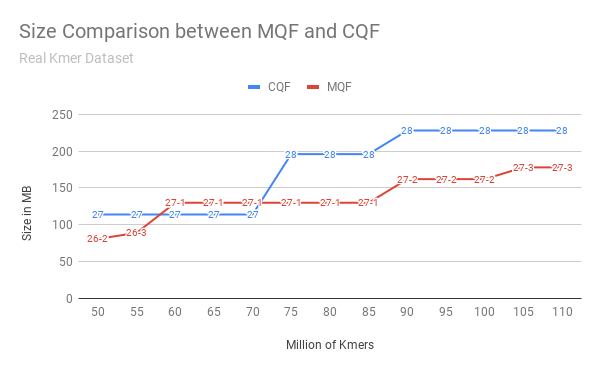


**Supplementary Figure 3: Detailed Memory Consumption Comparison for the real k-mers generated from ERR1050075.** The numbers on the CQF curve are the used “Q sizes”. While the numbers on MQF are “Q size-fixed counter size”.

**
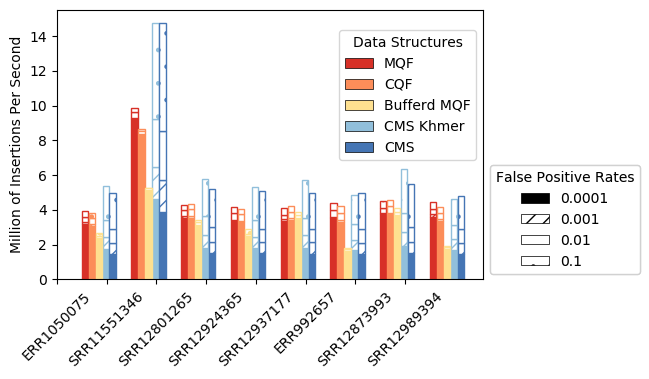
**

**Supplementary Figure 4: Insertions rate comparison of four data-structures in real datasets:** MQF, CQF, buffered MQF (using 1/3 the size of other structures), Khmer implementation of CMS, and original implementation of CMS. The performance is plotted as a bar graph where the pattern of each bar shows the cumulative increase in insertions for different false positive rates.


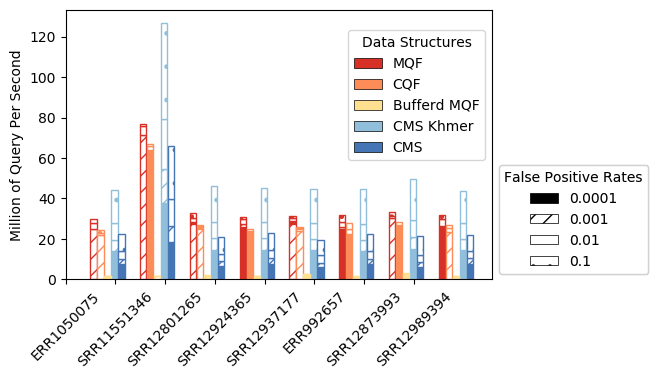


**Supplementary Figure 5: Query rate comparison of four data-structures in real datasets:** MQF, CQF, buffered MQF (using 1/3 the size of other structures), Khmer implementation of CMS, and original implementation of CMS. The performance is plotted as a bar graph where the pattern of each bar shows the cumulative increase in queries for different false positive rates.


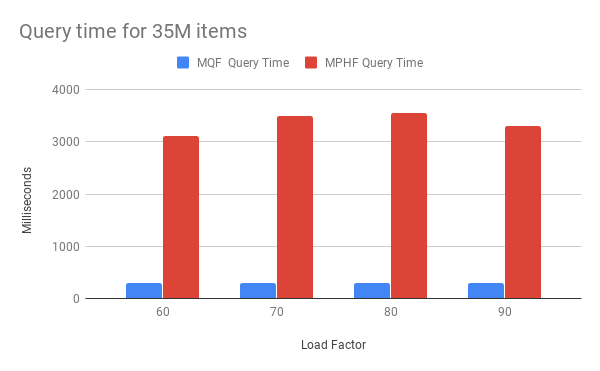


**Supplementary Figure 6: Query performance Comparison of MQF and MPHF**. The query times of 35M existed k-mers were measured for MQF structures with 60%, 70%, 80%, and 90% load factors and MPHF structures storing matching datasets.


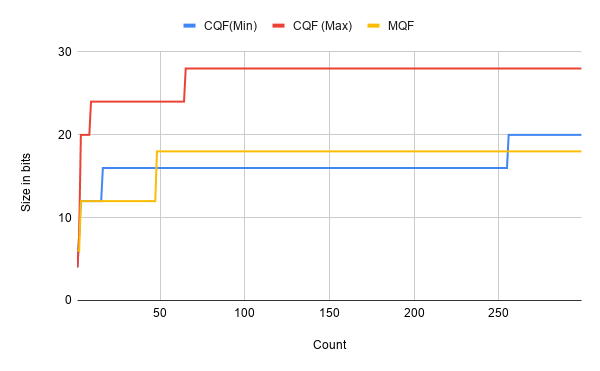


**Supplementary Figure 7**: **Number of slots required by CQF and MQF:** The number of slots needed by each data structure to represent a k-mer and its count. Since CQF needs to add special slots for some items CQF(Min) and CQF(MAX) curves are added to represent the minimum and the maximum number of slots needed by CQF. In this experiment, both data structures are using 4-bit total slot size. The MQF slot size includes the fixed-size counter.


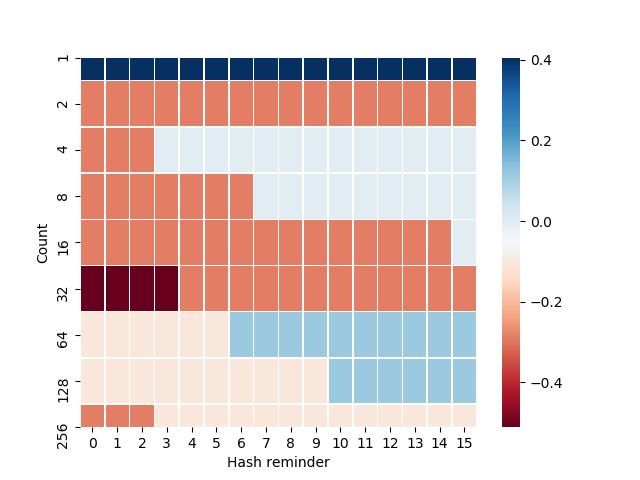


**Supplementary Figure 8: A comparison between the** **number of slots required by CQF and MQF to represent a k-mer and its count**. A dataset of 144 items was created in the form of k-mer and its count. All possible values of hash reminders of size 4 (16 items) are created with counts in the range starting from 1 to 256. Items were inserted into both data structures and the number of slots needed was recorded. Each cell in the figure displays the ratio of memory requirements on the log scale calculated as $log(\frac{(slot size * \#slots) in MQF}{(slot size * \#slots) in CQF})$ represented as a heat map where reddish shades mean that MQF needs fewer slots than CQF.


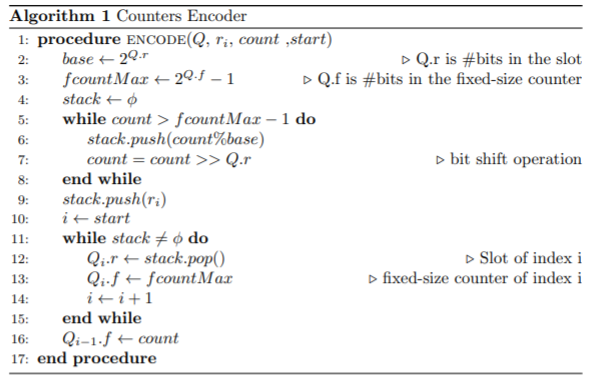


**Supplementary Figure 9: Counters encoder algorithm.** The Algorithm encodes the item and its count into one or more slots. The first slot is reserved for the item’s remaining, and the variable number of slots follows to encode the item’s count.

| **Distribution** | **CQF** | **MQF** | **Increased Capacity** |
| --- | --- | --- | --- |
| **Zipfian Z2** | 402 | 671 | 66.92% |
| **Zipfian Z3** | 161 | 161 | 0.00% |
| **Zipfian Z5** | 134 | 134 | 0.00% |
| **Uniform** | 402 | 1275 | 217.16% |
| **Simulated DNAseq** | 147 | 268 | 82.31% |
| **ERR1050075** | 402 | 671 | 66.92% |
| **SRR11551346** | 469 | 603 | 28.57% |
| **SRR12801265** | 234 | 369 | 57.69% |
| **SRR12924365** | 335 | 469 | 40.00% |
| **SRR12937177** | 177 | 275 | 55.37% |
| **ERR992657_2** | 134 | 201 | 50.00% |
| **SRR12873993** | 301 | 342 | 13.62% |
| **SRR12989394** | 295 | 348 | 17.97% |

**Supplementary Table 1:** Number of items (in millions) inserted to achieve a 90% load factor in compact hash tables.

| **Sample Accession** | **Description** |
| --- | --- |
| **ERR1050075** | PolyA selected mRNA sequencing of Homo sapiens |
| **SRR11551346** | Whole Exome sequencing of Homo sapiens |
| **SRR12801265** | 3' mRNA-Seq - Mouse B cell lymphoma |
| **SRR12924365** | Single-cell sequencing RNA of Mice |
| **SRR12937177** | RAD-Seq with Ion Torrent for Cucurbita argyrosperma subsp |
| **ERR992657** | DNA Whole-genome sequencing |
| **SRR12873993** | RNA immunoprecipitation sequencing (RIP-Seq) - human cells infected with Ebola virus |
| **SRR12989394** | DNase-seq of mitochondrial genome of Mukaria Splendida |

**Supplementary Table 3:** Description of all the samples used in the paper.
